# Supplementary figures and images for: TBK1 activity regulates the directionality of axonal transport of signalling endosomes
Source: Life Sci Alliance. 2026 Feb 4;9(4):e202503527. doi: 10.26508/lsa.202503527 (PMC12872394; doi:10.26508/lsa.202503527)

Panel C

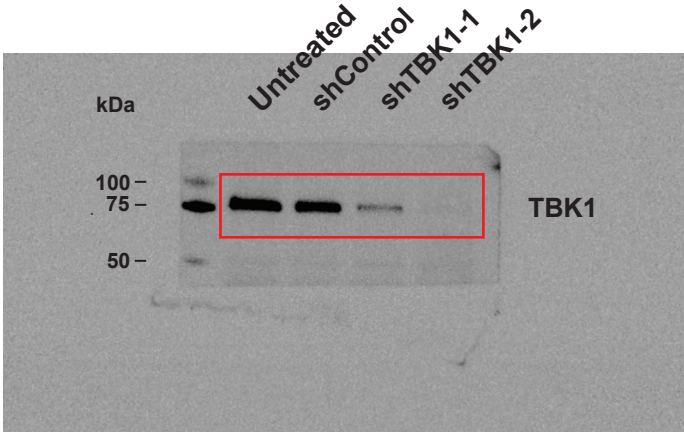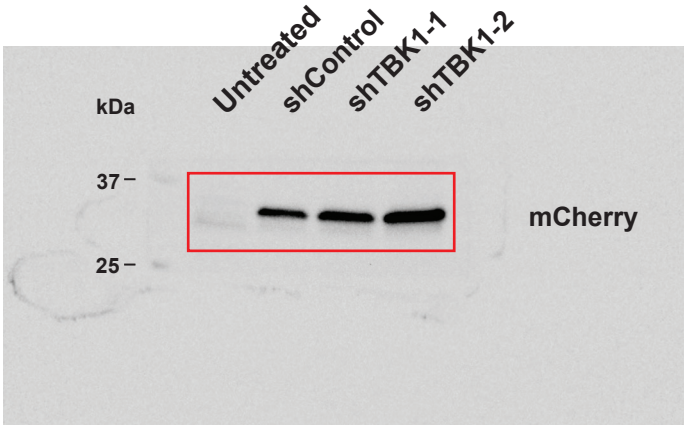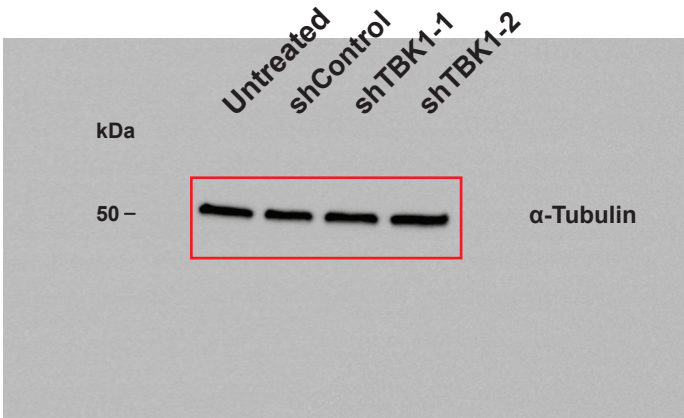

Supplement: Supplementary file 1 [file LSA-2025-03527_SdataFS1.pdf]

# SourceDataF4

## Panel A

Motor neurons

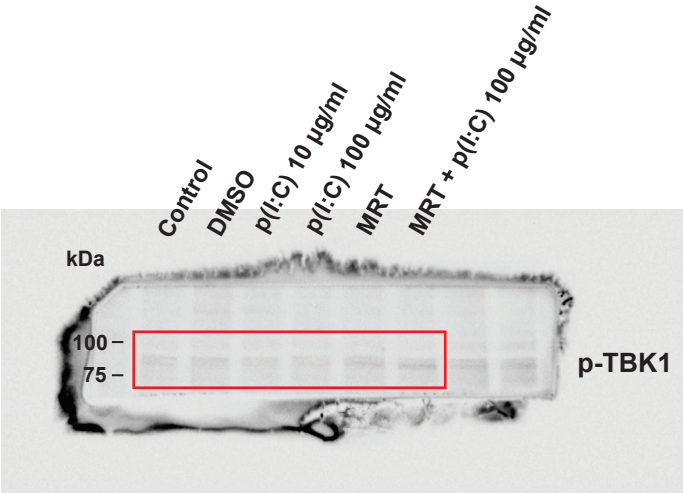

N2a cells

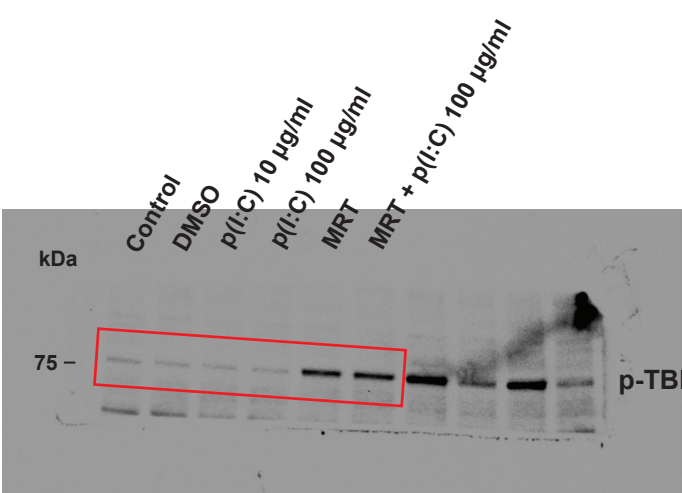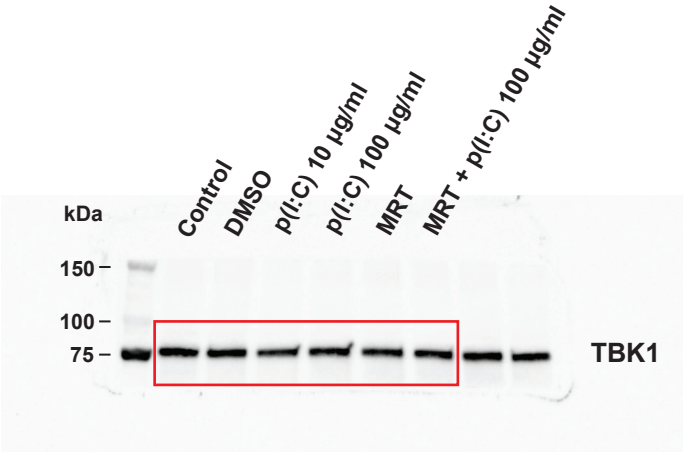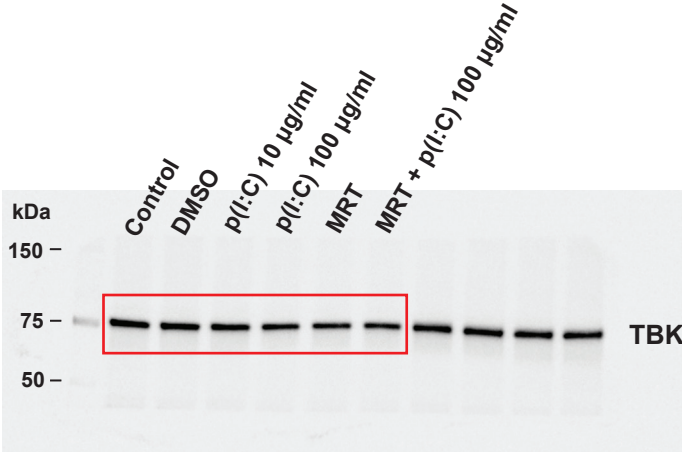

Supplement: Supplementary file 2 [file LSA-2025-03527_SdataF4.pdf]

Panel A

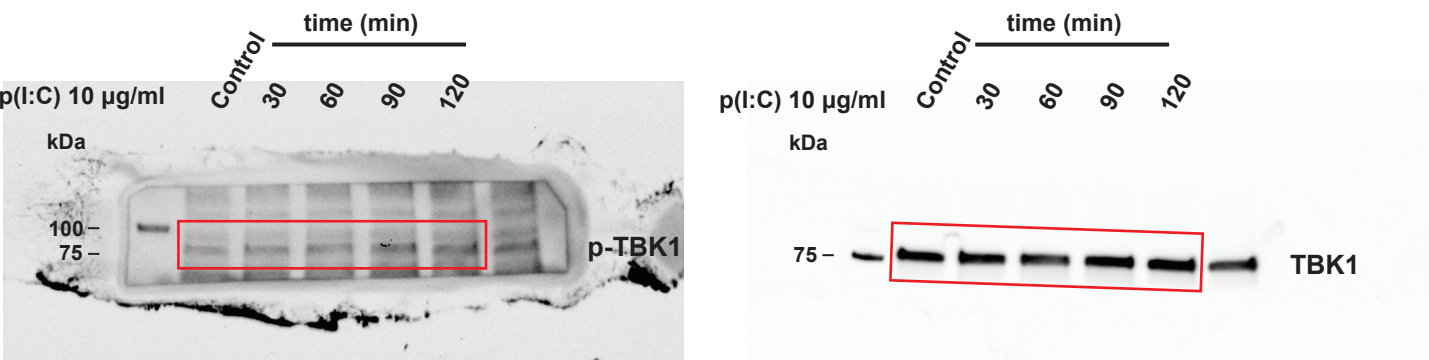

Panel C

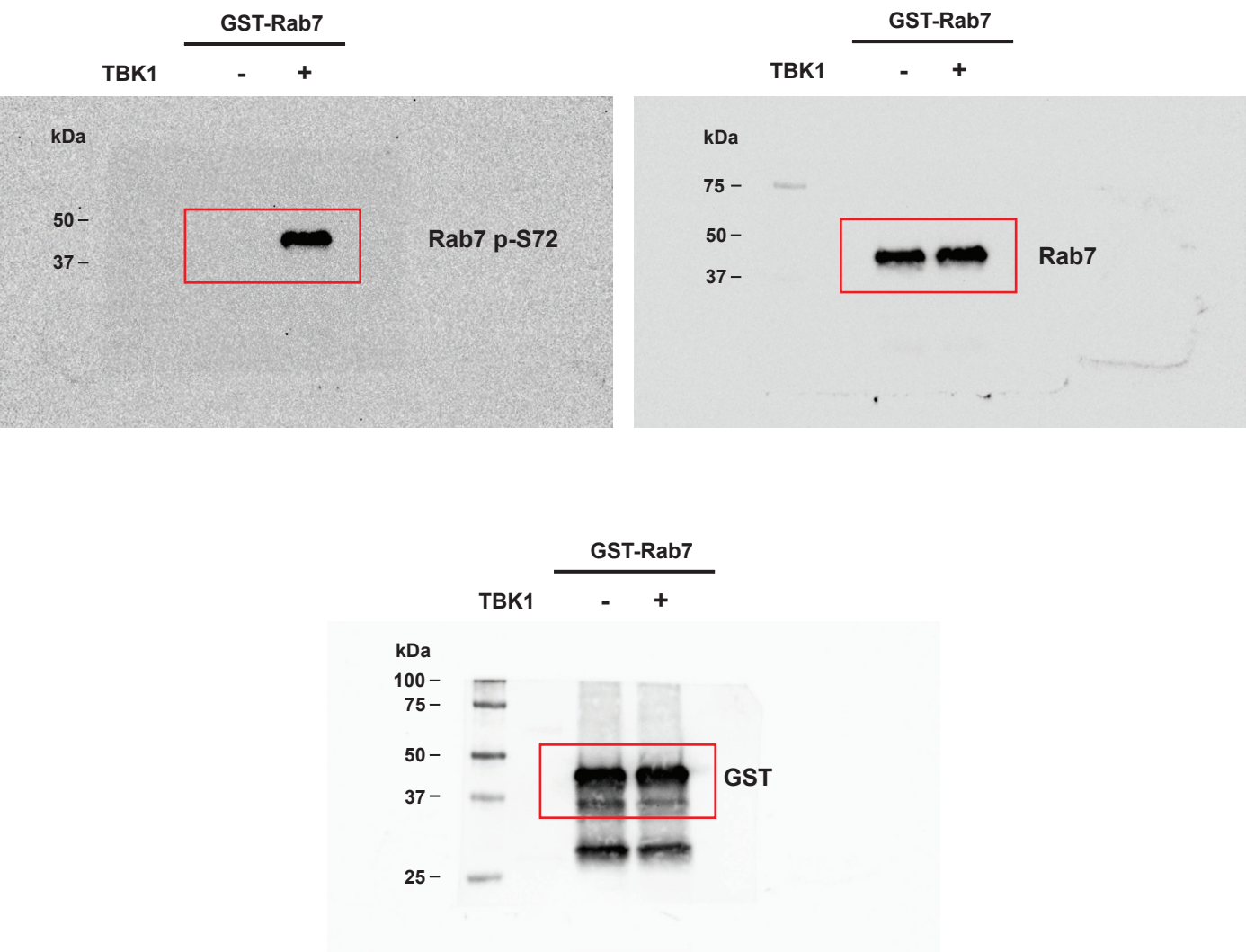

Supplement: Supplementary file 3 [file LSA-2025-03527_SdataFS4.pdf]
